# 2560. Individual Meropenem Epithelial Lining Fluid and Plasma PK/PD Target Attainment in Patients with Pneumonia

**DOI:** 10.1093/ofid/ofad500.2177

**Published:** 2023-11-27

**Authors:** Roxane Rohani, Paul R Yarnold, Marc H Scheetz, Michael N Neely, Mengjia Kang, Helen K Donnelly, Kay Dedicatoria, Sophia Nozick, Rachel Medernach, Alan R Hauser, Egon A Ozer, Estefani Diaz, Alexander V Misharin, Richard G Wunderink, Nathaniel J Rhodes

**Affiliations:** Chicago Medical School, Rosalind Franklin University of Medicine and Science, North Chicago, Illinois; Optimal Data Analysis LLC, Chicago, Illinois; Midwestern University, Downers Grove, Illinois; The Saban Research Institute, Children’s Hospital Los Angeles, University of Southern California, Los Angeles, CA, USA, Los Angeles, California; Northwestern University, Chicago, Illinois; Northwestern University, Chicago, Illinois; Midwestern University, Downers Grove, Illinois; Northwestern University, Chicago, Illinois; Northwestern University Feinberg School of Medicine, Chicago, Illinois; Northwestern University, Chicago, Illinois; Northwestern University Feinberg School of Medicine, Chicago, Illinois; Northwestern University, Chicago, Illinois; Northwestern University, Chicago, Illinois; Northwestern University Feinberg School of Medicine, Chicago, Illinois; Midwestern University, Downers Grove, Illinois

## Abstract

**Background:**

Whether pharmacokinetic (PK)/pharmacodynamic (PD) target attainment for meropenem measured in plasma is a clinically reliable surrogate for target attainment in the epithelial lining fluid (ELF) in pneumonia is unclear. The objective of the current study was to characterize target attainment in plasma and ELF using prospectively collected samples and to define predictors of optimal target attainment.

**Methods:**

Individual Bayesian meropenem plasma and ELF profiles were generated using Pmetrics for the first 24 hours of treatment. First 24-hr PK/PD target attainment was evaluated *vs*. PK/PD goals of 100% T_>1xMIC_ and 100% T_>4xMIC_ for a susceptible MIC of 2 mg/L.

**Results:**

Sixty-seven patients contributed ELF and plasma PK data (range: 1-3 samples/patient for each matrix). Mean population CL, V_plasma_, and V_ELF_ estimates were 6.2 L/hr, 45.7, and 28.3 L. For the 100% T_>4xMIC_ goal the following target attainment groups were identified: optimal (≥95%) in plasma and ELF (n=27/67, 40%), near-optimal ( >50% and < 95%) in both plasma and ELF (n=11/67, 16.4%), suboptimal (< 50%) in ELF only (n=15/67, 22.4%), suboptimal (< 50%) in plasma only (n=1/67, 1.5%), and suboptimal (< 50%) in both ELF and plasma (n=13, 19.4%). Loading doses significantly improved the likelihood of optimal *vs*. suboptimal target attainment in ELF and plasma (96.2% *vs*. 14.3%; *p*< 0.0001) but did not reliably discriminate other groups.

**Figure:**
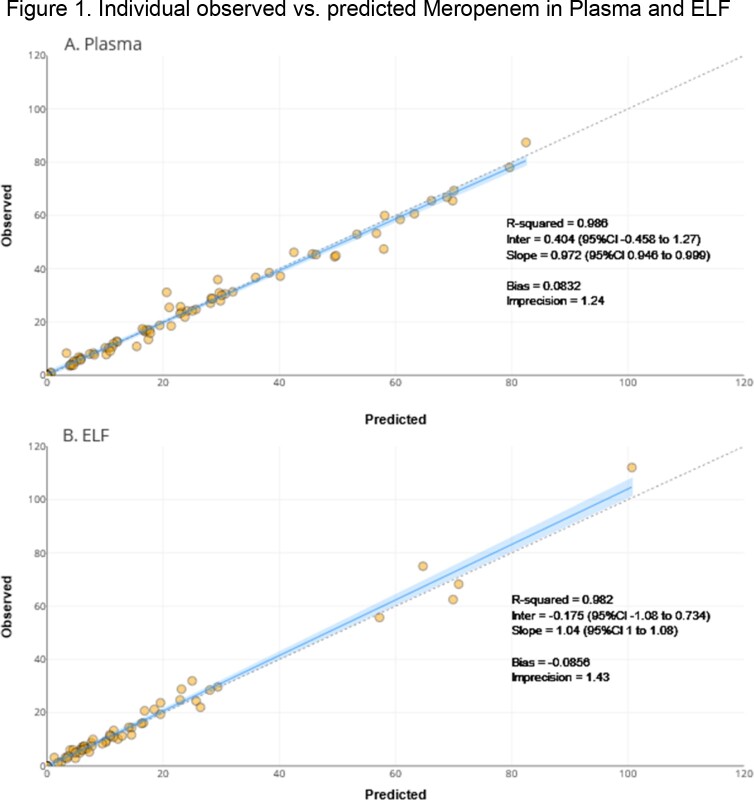

Individual observed meropenem (ordinate) and model predicted meropenem (abscissa) concentrations in plasma (A) and ELF (B) in patients with pneumonia.

**Figure:**
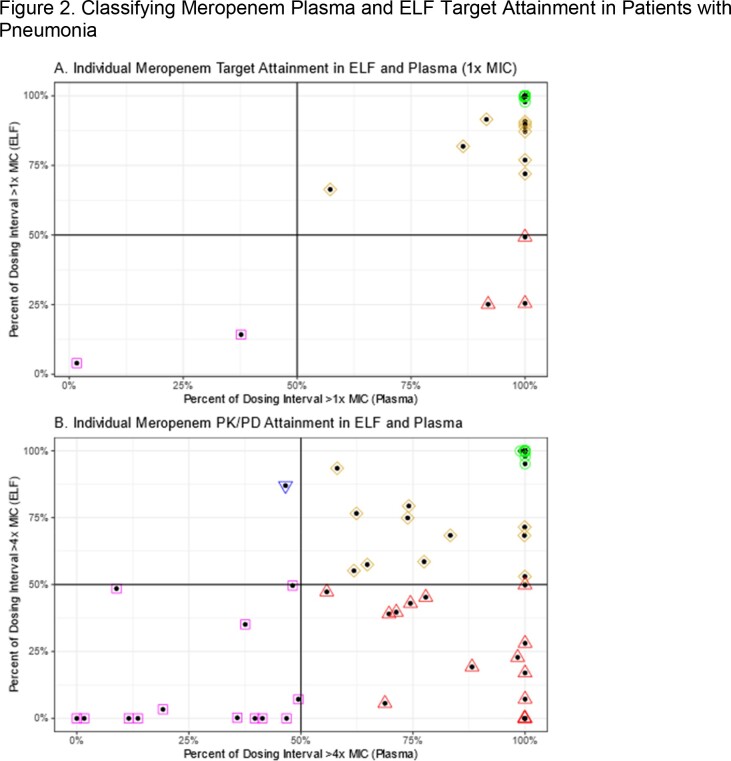

Panel A.) grouping according to a 1xMIC target. A total of 53 patients had optimal (green) attainment in both plasma and ELF, 9 patients had near optimal attainment in plasma and ELF (gold), 3 had suboptimal attainment in ELF only (red), and 2 had suboptimal attainment in both plasma and ELF (purple). Panel B.) grouping according to a 4xMIC target. A total of 27 patients had optimal (green) attainment in both plasma and ELF, 11 patients had near optimal attainment in plasma and ELF (gold), 1 patient had suboptimal attainment in plasma only (blue), 15 had suboptimal attainment in ELF only (red), and 13 had suboptimal attainment in both plasma and ELF (purple).

**Conclusion:**

Optimal target attainment was achieved in only 40% of patients while nearly 20% of patients had suboptimal attainment in both plasma and ELF. Use of plasma as a surrogate for ELF would miss up to 20% of patients with suboptimal ELF attainment, suggesting that therapeutic monitoring of ELF may be required in some patients to optimize PK/PD attainment.

**Disclosures:**

**Marc H. Scheetz, PharmD, MSc**, Abbvie: Advisor/Consultant|ASHP: Honoraria|Chambless, Higdon, Richardson, Katz & Griggs, LLP: Expert Testimony|Cidara: Advisor/Consultant|Entasis: Advisor/Consultant|F2G: Advisor/Consultant|GSK: Advisor/Consultant|Guidepoint Global: Honoraria|Hall, Booth, Smith, P.C.: Expert Testimony|Merck: Advisor/Consultant|Reminger Co., L.P.A: Expert Testimony|Spero: Advisor/Consultant|Takeda: Advisor/Consultant|Taylor, English, Duma, LLP: Expert Testimony|Third Pole Therapeutics: Advisor/Consultant **Michael N. Neely, MD**, Astellas Pharma Global Development, Inc.: Advisor/Consultant|Astellas Pharma Global Development, Inc.: Support for the present publication **Richard G. Wunderink, MD**, bioMerieux: Honoraria|Kariius: Clinical Evaluation Committee|LaJolla: Advisor/Consultant|Pfizer, Inc: Clinical Evaluation Committee|Shionogi: Advisor/Consultant **Nathaniel J. Rhodes, PharmD MS**, Third Pole Therapeutics: Advisor/Consultant

